# Microorganism Strains, Environmentally Friendly and Biological Preparations Against *Meloidogyne hapla* Chitwood, 1949 and Their Impact on Fruit Quality and Tomato Crop Structure

**DOI:** 10.3390/microorganisms12122586

**Published:** 2024-12-13

**Authors:** Svetlana Nikolaevna Nekoval, Arina Konstantinovna Churikova, Oksana Aleksandrovna Maskalenko, Zhanneta Zaurovna Tukhuzheva, Valentin Valentinovich Ivanov

**Affiliations:** Federal Research Center of Biological Plant Protection, p/o 39, 350039 Krasnodar, Russia; arina.churikova98@mail.ru (A.K.C.); d.o.a.123@mail.ru (O.A.M.); zhanneta2602@gmail.com (Z.Z.T.); ivanov.offiziell@gmail.com (V.V.I.)

**Keywords:** tomato, microorganisms, nematicides, root-knot nematodes, *Meloidogyne hapla*, biochemical parameters, yield, biological efficacy

## Abstract

The primary aim of this research was to study the effectiveness of various strains of antagonist microorganisms and biological preparations against *Meloidogyne hapla*, in addition to their impact on the quality of tomato fruits and crop structure. Four microorganism strains and three registered environmentally safe nematicides were used in the experiment presented herein. The results showed that the strains *Paecilomyces lilacinus* F-22BK/6 and *Arthrobotrys conoides* F-22BK/4 had the greatest biological efficacy, reducing the number of galls on tomato plants by 91.8% and 88.4%, values comparable with the results of the chemical control Vydate 5G. The *Metarhizium anisopliae* F-22BK/2 and *Arthrobotrys conoides* F-22BK/4 treatments showed the best results, increasing the fruit weight by 8.6% and 9.9%, in addition to increasing the tomato yield by 5.0% and 13.3%. These strains contributed to an increase in sugar content, whereas the concentration of vitamin C was reduced in the *Trichoderma viride* F-294 and Fitoverm treatments, indicating a high level of oxidative stress in the latter treatments. The results of this study confirm the prospects of using biological nematicides against phytoparasitic nematodes, which will not only enable effective control of their population but also improve the quality of agricultural products, minimizing harm to the environment and human health.

## 1. Introduction

Providing high-quality and sufficient agricultural products to the world’s population is one of the most pressing issues today [1]. The human diet cannot be imagined without vegetables. The world’s population is increasing year by year, leading to an increased need to grow vegetable crops in large quantities [1,2]. In order to preserve, increase, and improve the quality of agricultural products, it is necessary to pay particular attention to cultivation technologies and plant protection systems that can be used to protect against harmful effects [2,3].

Tomato (*Solanum lycopersicum* L.) is one of the most commonly consumed vegetables worldwide. The main bioactive compounds in tomatoes are sugars, acids, lycopene, glutamic acid, and vitamin C. The effects of these compounds on human health have been extensively studied [4]. Both fresh and processed tomatoes are widely used in many dishes. Tomato varieties and hybrids with improved organoleptic properties are especially valued. High sugar contents and relatively high acid contents improve the taste of the fruit [5]. However, different cultivation methods significantly affect the yield and quality of tomatoes [6].

Significant yield losses and a decrease in the quality of tomato fruits occur due to the spread of and damage caused by phytoparasitic nematodes, including root-knot nematodes, namely *Meloidogyne* spp. These nematodes can reduce crop yields by up to 30% and, in some cases, even greater amounts [7,8].

The damage caused by the root-knot nematodes (*Meloidogyne* spp.) may impact the availability of food. They are widespread, obligate endoparasites of the plant root system, whose survival and reproduction are entirely dependent on the host [9]. Worldwide, there are more than 100 species of root-knot nematodes on more than 3000 host plants, including vegetable crops [10,11]. Invasion by these phytoparasites makes the plant more susceptible to bacterial and fungal diseases [12,13].

Tomatoes have an innate immune system that reacts to danger signals and triggers protective reactions, including the release of reactive oxygen species (ROS) [14]. Ascorbic acid is an antioxidant that effectively neutralizes ROS and is involved in protecting plant cells against oxidative stress [15,16]. Sugars provide energy and structural material for plant defense responses, and they can also act as signaling molecules that interact with the hormonal signaling network that regulates the plant immune system [15].

More recently, the use of rational, environmentally friendly plant protection products has attracted more attention from farmers as a safer alternative to chemical products. These products, in addition to protective mechanisms, can have a positive effect on plant growth and development [17]. The development of alternative crop protection methods is becoming increasingly important for the control of root-knot nematodes, as there are many restrictions on the use of chemical nematicides due to their detrimental effects on the environment and human health [17,18,19].

A significant number of biological nematicides have been developed and have become commercially available to farmers in India, Egypt, China, Germany, Colombia, the Philippines, and other countries [20,21].

In Russia, the only currently used biological nematicide is Nematophagin–Mycopro, which is a mycelial suspension of the predatory nematophagous fungus *Arthrobotrys oligospora* F-1303 [19,22]. This preparation is registered and included in the Directory of Pesticides and Agrochemicals Permitted for Use in the Russian Federation. This list is also supplemented with three more chemical nematicides based on the active ingredients oxamyl (Vydate 5G and Palitsa) and fluopyram (Verango). At the end of December 2023, the registration period for the preparation based on avermectin C (Fitoverm) expired [23].

The aim of this research was to study the biological efficacy of microorganism strains and environmentally friendly and biological preparations against the northern root-knot nematode and to assess their impact on the quality of fruits and the structure of tomato crops infected with *Meloidogyne hapla* Chitwood, 1949.

## 2. Materials and Methods

### 2.1. Research Location

Experimental studies were carried out at the Laboratory of Bio-rational Means and Technologies for Plant Protection for Environmentally Friendly, Resource-Saving, and Organic Agriculture and in the greenhouse of the Federal State Budgetary Scientific Institution “Federal Research Center of Biological Plant Protection” (FSBSI FRCBPP), Krasnodar, Russia.

### 2.2. Research Objects

The most common species of root-knot nematode in the south of Russia was selected for the growth experiment. The northern root-knot nematode (*M. hapla*) was maintained and propagated on susceptible tomato plants in growing pots [19].

In the experiment, we used liquid cultures of the microorganism strains, antagonists of root-knot nematodes, sourced from the working collection of Biotekhagro LLC (Timashevsk, Russia) (Table 1).

A Nematophagin–Mycopro preparation was used as a biological control; for the chemical control, we used a Vydate 5G preparation and a Fitoverm preparation, which, at the time of the experiment, were included in the “Directory of Pesticides and Agrochemicals Permitted for Use in the Russian Federation, 2023” (Table 2) [23].

When assessing the biological efficiency of microorganism strains from the working collection of Biotekhagro LLC, in addition to environmentally friendly preparations against the background of artificial contamination of the soil with the northern root-knot nematode, their effect on the fruits of the tomato variety Ranniy-83 was studied.

Since 2015, the Ranniy-83 variety has been included in the State Register of the Russian Federation for cultivation in the field and under film shelters on private farms. Ranniy-83 is an early-ripening variety and is suitable for salads. The plant is determinate, and its leaves are medium-sized and green in color. The inflorescence is of medium size and articulated. The fruit is flat and round, loose, and slightly ribbed. The color of the unripe fruit is green; in comparison, the ripe fruit is red. It is resistant to tobacco mosaic virus and fusarium but susceptible to infection by root-knot nematodes [24].

### 2.3. Artificial Infection of Tomato Plants with the Northern Root-Knot Nematode

Tomato plants were inoculated with a suspension of the root-knot nematode at a rate of 5000 infective juveniles per 1 L of soil [25]. Egg sacs of the *M. hapla* were removed from the stock culture (tomato). Under a binocular magnifying glass, the egg sacs were separated from the roots with dissecting needles and transferred to Petri dishes with water, stored at room temperature (25 ± 3 °C), and the water volume was regularly aerated with a pipette. After 3 days, the larvae that hatched from the eggs were collected with a pipette and placed into a test tube for further examination in a greenhouse [19].

Based on different conditions, the growth experiments were conducted in the greenhouses of FSBSI FRCBPP as follows:

1. Control (without treatment; watering);
2. Biological control (Nematophagin–Mycopro (*Arthrobotrys oligospora* F-1303));
3. Chemical control (Vydate 5G (Oxamyl, 50 g/kg));
4. Fitoverm (Aversectin S, 8 g/kg);
5. *Metarhizium anisopliae* F-22BK/2;
6. *Arthrobotrys conoides* F-22BK/4;
7. *Paecilomyces lilacinus* F-22BK/6;
8. *Trichoderma viride* F-294.

The biological efficacy of liquid cultures of microorganism strains and nematicides against the background of artificial infection of the soil with *M. hapla* was determined in growing pots (5 l seedling pots) on the susceptible tomato variety Ranniy-83 [19,26]. When preparing the working solution, 150 mL of water and 50 mL of each liquid culture (in a ratio of 3:1) were used and added to the soil previously infected with *M. hapla*.

Nematicides were added to the soil according to the declared application rates [23]. The preparations Vydate 5G, Fitoverm, and Nematophagin–Mycopro were applied dry with immediate incorporation into the soil to a depth of 5–10 cm.

Four days after treatment with liquid cultures of microorganisms and application of nematicides to the soil, 50-day-old tomato seedlings of a variety susceptible to the northern root-knot nematode were planted in all experimental cases. The control was the treatment with untreated soil.

Biological efficiency against *M. hapla* was determined 180 days after tomato planting by the number of galls on one plant using the formula of Abbott, 1925 [19]:

BE = 100 × (1 − A/B)(1)

where: BE—biological efficacy or mortality rate of individuals, %; A—the number of individuals after treatment; B—the number of individuals in the control (without treatment).

Workflow: 1 susceptible variety and 8 experimental treatments repeated 10 times.

In the greenhouse, the optimal temperature of 23 to 25 °C was maintained, with a relative air humidity of 60% and uniform soil moisture.

### 2.4. Assessment of the Effect of Microorganisms and Environmentally Friendly Biological Nematicides on the Quality of Fruits and the Structure of the Tomato Crop When Infected with M. hapla

The quality of fruits and the structure of the tomato crop when assessing the effect of microorganisms and environmentally friendly biological nematicides on plants infected with *M. hapla* were determined according to Russian GOSTs (state standards), namely GOST 24556-89 “Processed fruit and vegetable products. Methods for determining vitamin C” [27,28].

The concentration of sugars in the tomato fruits was determined using the refractometric method. For this purpose, from 0.05 to 0.5 g of tomato juice was used, and measurements were taken on the device’s scale [29].

### 2.5. Statistical Analysis

Statistical data processing was performed using standard methods with Microsoft Office Excel 2007 and the one-way analysis of variance (ANOVA). The data were shown as the average of ten repeated samples ± standard deviation (SD). Duncan’s criterion was used, and differences were considered statistically significant at *p* < 0.05. Spearman’s rank correlation coefficients were used to determine the relationships between biochemical parameters in the tomato fruits (*p* < 0.05).

## 3. Results

### 3.1. The Effect of Microorganisms and Environmentally Friendly and Biological Nematicides on the Development of Tomato Plants Under Conditions of Artificial Infection with M. hapla

The assessment of the biological efficacy (BE, %) of microbial agents and environmentally safe preparations against the background of artificial infection of the soil with the northern root-knot nematode was carried out on the susceptible tomato variety Ranniy-83 (Table 3).

Soil treatment in growing pots resulted in a decrease in the number of galls per plant. The results of the experiment showed that the strains of microorganisms and environmentally safe preparations used reduced the infection rate of the susceptible tomato variety Ranniy-83 with the northern root-knot nematode by 48.0–96.8%.

The Duncan criterion allows us to determine which of the studied treatments significantly differ from each other in terms of the studied indicators; as a result of the test, the average values of groups that do not have statistically significant differences are combined into groups denoted by the same letters.

The results of the Duncan criterion showed that the strains *Paecilomyces lilacinus* F-22 BK/6 and *Arthrobotrys conoides* F-22BK/4 showed no significant differences in biological efficacy when compared with one another but significantly exceeded the control group. The highest biological efficacy was recorded in *Paecilomyces lilacinus* F-22BK/6 strains (91.8%), which was in the same group as the Vydate 5G chemical control (96.8%). Treatments using *Trichoderma viride* F-294 and Fitoverm showed lower efficiency and did not differ from each other; however, they were significantly inferior to the above-mentioned treatments.

The best biological efficacy was noted when using a chemical control (96.8%). The use of *Paecilomyces lilacinus* F-22BK/6, *Arthrobotrys conoides* F-22BK/4, and *Metarhizium anisopliae* F-22BK/2 strains also made it possible to obtain high biological efficacy rates, wherein there were 6.2–10.4 galls/plant.

### 3.2. Effect of the Employed Methods for M. hapla Population Control on Tomato Crop Structure

A positive effect of microorganism strains and promising environmentally safe biological preparations on the structure of tomato crops was noted (Table 4).

In the Ranniy-83 variety, the average fruit weight was higher than in the control in treatments using the biological control and the microorganism strains *Metarhizium anisopliae* F-22BK/2, *Arthrobotrys conoides* F-22BK/4, *Paecilomyces lilacinus* F-22BK/6, and *Trichoderma viride* F-294. Similar results were obtained during harvesting, where the value of the preserved yield ranged from 5.0 to 13.3%.

### 3.3. Effect of M. hapla Population Control Agents on Biochemical Indicators of Tomato Fruits

**Figure 1:**
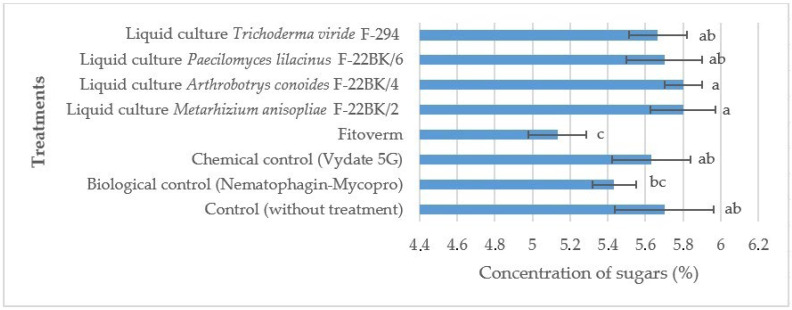
Effect of the studied preparations and liquid cultures of microorganism strains on the concentration of sugars in the fruits of the tomato variety Ranniy-83 when infected with the *M. hapla*. The average values with the same letter do not differ significantly.

It was found that in the treatments with *M. anisopliae* F-22BK/2 and *A. conoides* F-22BK/4, there was an increase in sugar content in the tomato fruits. In the treatments with *P. lilacinus* F-22BK/6 and *T. viride* F-294, the sugar concentration was at the level of the control (without treatments). In the other experimental treatments, there was a 1.8–10.5% decrease in sugar content. When repeatedly comparing the average values between the experimental groups, it was found that the data marked by the same letter did not differ significantly (Figure 1).

**Figure 2:**
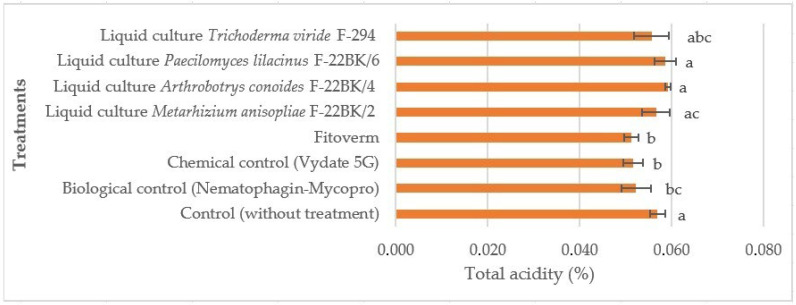
Effect of the studied preparations and liquid cultures of microorganism strains on the total acidity in the fruits of the tomato variety Ranniy-83 when infected with the *M. hapla*. The average values with the same letter do not differ significantly.

Attention should be paid to the treatments using the fungal strains *P. lilacinus* F-22BK/6, *M. anisopliae* F-22BK/2, *A. conoides* F-22BK/4, and *T. viride* F-294, in which the total acid content was either higher than the control or equal to it. These data suggest that the optimal combination of total acidity in relation to sugars makes the variety the most delicious (Figure 2).

For statistical data processing, one-factor analysis of variance (ANOVA) was used, which revealed significant differences between the empirical variables (*p*-value = 0.0041, indicating a significance level of *p* < 0.05). Empirical variables marked with the same letters do not show statistically significant differences when compared to one another. The control treatment (without treatments) showed the lowest acidity in tomato fruits (marked “a”), which indicates no effect on plant metabolism. The biological control, the chemical control, and the Phytoverm preparation demonstrated similar levels of total acidity, indicated by the letter “b”, which indicates some changes in the metabolism of plants under their influence. The highest acidity in fruits was observed in plants treated with liquid cultures of *T. viride* F-294 and *M. anisopliae* F-22BK/2 (marked “abc” and “ac”).

**Figure 3:**
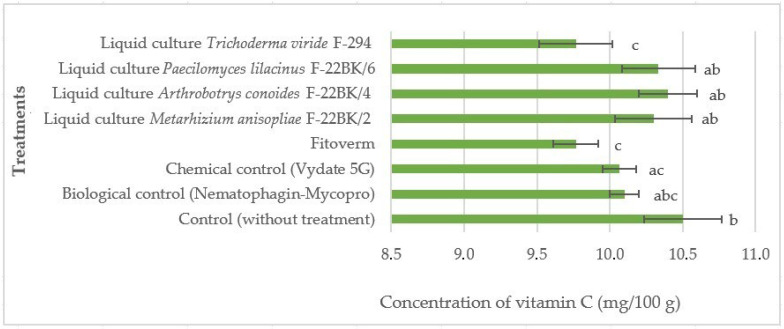
Effect of the studied preparations and liquid cultures of microorganism strains on the vitamin C content in the fruits of the tomato variety Ranniy-83 when infected with the *M. hapla*. The average values with the same letter do not differ significantly.

When using the Fitoverm preparation and the liquid culture of the *Trichoderma viride* F-294 strain, a clear decrease of 0.3–0.6% in the content of ascorbic acid in tomato fruits was observed in comparison with the other experimental treatments. This result may indicate a high level of oxidative stress in plants in these experimental treatments, which in turn leads to a decrease in the concentration of vitamin C (Figure 3).

The obtained *p*-value was 0.0028, which indicates the presence of statistically significant differences between the groups at a significance level of *p* < 0.05. The control treatment (without treatments) showed one of the highest concentrations of vitamin C in the tomato fruits (roughly 10.5 mg/100 g), indicated by the letter “b”, which indicates significant differences compared to the other treatments. High levels of vitamin C indicate normal plant metabolism, which has not been disrupted by external factors or treatments.

The biological control (Nematophagin–Mycopro) and the chemical control (Vydate 5G) provided similar results (about 10.1 mg/100 g, marked “abc” and “ac”), indicating a minimal effect of these preparations on plant metabolism. The Phytoverm preparation showed a decrease in the concentration of vitamin C in the tomato fruits (about 9.8 mg/100 g, marked “c”), which may indicate a stressful effect on the plants. Liquid cultures of microorganisms such as *M. anisopliae* F-22BK/2 and *A. conoides* F-22BK/4 contributed to maintaining high levels of vitamin C (roughly 10.1–10.6 mg/100 g, marked “ab”), which may indicate their positive effect on plant metabolism.

### 3.4. Correlation Analysis in Measuring Biochemical Indicators in Tomato Plants

To assess the impact of the treatments, a correlation analysis of the biochemical indicators of tomato fruits after treatment with all treatments of the preparations was performed. We calculated the linear correlation coefficients, and the results of the analysis are presented below in the form of a table (Table 5).

In the control, a weak positive correlation (0.14) between the content of sugars and acids indicates a minimal effect of stress caused by nematode infection on the metabolism of carbohydrates and organic acids. However, when using preparations such as Vydate 5G, a strong negative correlation (−0.97) may be associated with the increased use of carbohydrates (sugars) to maintain plant metabolism under stress, which reduces their availability for acid synthesis.

Negative correlations between sugar and vitamin C concentrations, especially pronounced in treatments using Phytoverm (−0.98) and Nematophagin–Mycopro (−0.97), may be associated with the metabolic redistribution of carbohydrates against the background of an enhanced antioxidant response. The positive correlation observed in treatments using the liquid culture of *Arthrobotrys conoides* F-22BK/4 (0.87) indicates a reduction in stress, which allows plants to distribute carbohydrates in a balanced manner to meet their energy needs and perform ascorbic acid synthesis.

Strong negative correlations, such as when using a liquid culture of *Paecilomyces lilacinus* F-22BK/6 (−0.59), may indicate a redistribution of resources in favor of antioxidant functions, which limits the accumulation of organic acids under severe stress. Treatments using liquid culture of *Trichoderma viride* F-294 (−0.38) also demonstrate a decrease in acid levels with an increase in vitamin C levels, which may be the result of activation of an antioxidant reaction to the detriment of acid synthesis.

## 4. Discussion

Liquid cultures of microorganism strains antagonistic to the northern root-knot nematode (*M. hapla*); *Metarhizium anisopliae* F-22BK/2; *Arthrobotrys conoides* F-22BK/4; *Paecilomyces lilacinus* F-22BK/6; and *Trichoderma viride* F-294, and environmentally friendly biological preparations (Nematophagin–Mycopro, Vydate 5G, and Fitoverm) were assessed in the present study. This study was carried out in a laboratory under the conditions of a growth experiment involving plants of the susceptible tomato variety Ranniy-83.

The microorganisms used suppress the development and spread of nematodes, a process that neutralizes their negative impact on the plant.

Our research allows us to conclude that, after treatment with liquid cultures of microorganisms and the introduction of nematicides into the soil, with tomato seedlings being planted 4 days later, it was possible to increase the crop yield, improve the biochemical indicators in the fruits, and reduce the damage caused by nematodes. An extensive study of the effect of biological nematicides, liquid cultures of antagonist microorganism strains, namely *Metarhizium anisopliae* F-22BK/2, *Arthrobotrys conoides* F-22BK/4, *Paecilomyces lilacinus* F-22BK/6, and *Trichoderma viride* F-294 as biological protection agents for tomato, was carried out, and their positive effect on the crop was determined.

Soluble sugars constitute the majority of tomato dry matter, and their content is an important indicator of the taste characteristics of tomato fruits [30]. Root-knot nematode infection triggers the immune response of plants, which is followed by the initiation of defense reactions that affect the content of sugars and secondary metabolites [16]. To analyze the response and the effect of infection on plant metabolism, we measured the concentrations of total acids, vitamin C, and sugars in the control and experimental treatments.

The results of studies by Kassam R. et al. (2022) confirm the ability of the *Metarhizium anisopliae* fungus to reduce the development of *Meloidogyne* spp. on tomato plants by 82.3% [31]. It has been demonstrated that the fungi *Metarhizium anisopliae* and *Paecilomyces lilacinus*, in addition to their positive effect on root-knot nematodes, significantly improve plant growth and yield indicators [32].

In the course of the growth experiment, the use of a liquid culture of the *Paecilomyces lilacinus* strain F-22BK/6 allowed us to reduce the number of galls on a tomato plant by 91.8%, which is only 5.0% lower than the results in the treatment with the chemical control Vydate 5G. When introducing this strain, we obtained a 10.0% increase in yield with improved taste qualities.

After studying the efficacy of five biopreparations, including preparations based on *Trichoderma*, *Metarhizium*, and other microorganisms, against a widespread *Meloidogyne* species, researchers from India found that biopreparations with an application rate of 3 g/kg of soil increase plant growth and reduce the nematode population in the field [17]. The results of a study by Kumari M. et al. (2020) showed that the introduction of the *Trichoderma viride* fungus into the soil with application rates of 10 and 15 mL/kg of soil significantly affected the growth of tomato plants and reduced the reproduction rate of root-knot nematodes [33].

In our study, liquid cultures of the microorganism strains *Metarhizium anisopliae* F-22BK/2 and *Trichoderma viride* F-294, introduced into soil infected with *M. hapla* before planting tomato seedlings at a ratio of 150 mL of water/50 mL of liquid culture, allowed us to achieve biological efficacy within 86.2% and 65.8%, in addition to a yield increase of 5.0% and 8.3% compared to the control. In addition, liquid cultures had a positive effect on the sugar concentration and total acid content of the tomato plants. When using a liquid culture of the *Trichoderma viride* F-294 strain, a decrease in ascorbic acid content was observed compared to the other experimental treatments.

Through their two-year experiments, Soliman M. S. et al. (2021) showed that the predatory fungus *Arthrobotrys oligospora* significantly suppresses gall formation on tomato plants, reducing the number of nematodes at different stages of development, with it having a noticeable effect on plant growth [26]. The use of the *Arthrobotrys conoides* strain F-22BK/4 in our studies showed similar results in the growth experiment on tomato plants susceptible to *M. hapla*. The biological efficacy of this treatment was 88.4%, which is close to the indicators in the treatment with *Paecilomyces lilacinus* F-22BK/6.

In the experiments of Massoud M. A. et al. (2023), the effect of various formulations of abamectin (a chemical class of avermectins) was studied, where the total population density of root-knot nematodes decreased by 39.2–87.1%; in some cases, the use of abamectin had a negative effect on the growth of the studied crop [34]. Researchers from Italy found that the active substances abamectin and oxamyl increased tomato yield and reduced gall formation. Concurrently, it was noted that when used separately, their efficacy was not as high as in combination with fosthiazate, which is the active compound of chemical nematicides [35].

In our experiments, the preparations Fitoverm, based on avermectin C (chemical class avermectins), and Vydate 5G, based on oxamyl (chemical class carbamates), showed different results. The introduction of the Fitoverm preparation with immediate incorporation into the soil to a depth of 5–10 cm in dry form allowed for gall formation to be reduced by 58.8%, which is 38.0% lower than with the introduction of the Vydate 5G preparation. When measuring the indicators of the crop structure of the tomato variety Ranniy-83, no increase in yield was noted. The Fitoverm preparation reduced the content of ascorbic acid in comparison with the other experimental treatments. In the treatment with the Vydate 5G preparation, similar results were not noted.

The relationship between sugar concentration and total acidity in tomato fruits can be explained by a number of biochemical and physiological processes. Positive correlations, for example, in the case of using a liquid culture of the fungus *Paecilomyces lilacinus* F-22BK/6 (0.6), may be due to the fact that its active metabolites reduce stress and stimulate the balanced use of sugars and acids.

Under stress conditions caused by infection with root-knot nematodes, plants mobilize sugars to maintain energy metabolism and stimulate the respiratory process through the pathway of the pentose phosphate cycle, which is actively involved in stressful situations [36]. However, glucose serves as the starting material for the synthesis of vitamin C [37]. In conditions where sugar is actively used to maintain vital functions, its deficiency limits the synthesis of vitamin C, which leads to strong negative correlations.

The positive correlations between total acidity and vitamin C concentration in the control treatment (0.48) and when treated with a chemical control (0.85) can be explained by the interrelated biochemical pathways of the synthesis of organic acids and vitamin C. Ascorbic acid is involved in the metabolism of organic acids such as citric and malic acid, which are important for maintaining cell pH and other processes [38].

## 5. Conclusions

The results of this study on the biological efficacy of the aforementioned control agents against *M. hapla* in a greenhouse in growing pots made it possible to identify the most effective strains—*Paecilomyces lilacinus* F-22BK/6, *Arthrobotrys conoides* F-22BK/4, and *Metarhizium anisopliae* F-22BK/2—for the development of new nematicides against root-knot nematodes based on their attributes.

The use of antagonist microorganism strains and registered nematicides allowed us to achieve high efficiency in reducing the damage caused by *M. hapla*, in addition to improving the quality and yield of tomatoes, which emphasizes the prospects for their use in environmentally friendly plant protection systems. The data presented herein are an important contribution to the development of biological plant protection strategies and can be used to develop new nematicides and, subsequently, improve agricultural technologies.

## Figures and Tables

**Table 1:** Strains of microorganism antagonists of root-knot nematodes used in the study.

Microorganism Species	Strain	Titer, CFU/mL
*Metarhizium anisopliae*	F-22BK/2	(2.5 ± 0.1) × 10^7^
*Arthrobotrys conoides*	F-22BK/4	(3.5 ± 0.7) × 10^6^
*Paecilomyces lilacinus*	F-22BK/6	(2.0 ± 0.5) × 10^7^
*Trichoderma viride*	F-294	(4.0 ± 0.2) × 10^6^

**Table 2:** Chemical and biological controls in the 2023 growth experiment.

Preparation	Active Compounds	Chemical Class	Titer, CFU/mL
Nematophagin–Mycopro	*Arthrobotrys oligospora* F-1303	-	(3.0 ± 0.5) × 10^6^
Vydate 5G	Oxamyl	Carbamates	-
Fitoverm	Aversectin C	Avermectins + biological pesticides	-

**Table 3:** Effect of control treatments and liquid cultures of microorganism strains on the development of galls on the roots of variety Ranniy-83 in a greenhouse of the FSBSI FRCBPP, 2023.

Treatments	Number of Examined Plants, Pieces	Number of Infected Plants, Pieces	Number of Galls/Plant	Biological Efficacy, % *
Control (without treatment)	10	10	75.2 ± 1.2	-
Biological control (Nematophagin–Mycopro)	10	8	39.1 ± 0.7	48.0 ^c^
Chemical control (Vydate 5G)	10	1	2.4 ± 0.3	96.8 ^d^
Fitoverm	10	8	31.0 ± 0.5	58.8 ^a^
Liquid culture *Metarhizium anisopliae* F-22BK/2	10	5	10.4 ± 0.6	86.2 ^ab^
Liquid culture *Arthrobotrys conoides* F-22BK/4	10	5	8.7 ± 0.3	88.4 ^b^
Liquid culture *Paecilomyces lilacinus* F-22BK/6	10	4	6.2 ± 0.2	91.8 ^e^
Liquid culture *Trichoderma viride* F-294	10	6	25.7 ± 1.6	65.8 ^f^

* Average values with the same letter are not significantly different (*p* > 0.05).

**Table 4:** Structure of the tomato crop of the Ranniy-83 variety when using strains of microorganisms and promising, environmentally safe, biological preparations.

Treatments	Fruit Weight, g *	Yield, kg/m^2^	
		Average	Control Gain, %
Control (without treatments)	85.6 ^c^	6.0	-
Biological control (Nematophagin-Mycopro)	92.2 ^abc^	6.6	10.0
Chemical control (Vydate 5G)	83.1 ^ac^	5.5	-
Fitoverm	85.2 ^c^	5.8	-
Liquid culture *Metarhizium anisopliae* F-22BK/2	93.0 ^ab^	6.3	5.0
Liquid culture *Arthrobotrys conoides* F-22BK/4	94.1 ^ab^	6.8	13.3
Liquid culture *Paecilomyces lilacinus* F-22BK/6	90.3 ^ab^	6.6	10.0
Liquid culture *Trichoderma viride* F-294	87.4 ^c^	6.5	8.3

* The data represent the average weight of 10 tomato fruits. Average values with the same letter do not differ significantly (*p* < 0.05).

**Table 5:** Spearman’s rank correlation coefficients between biochemical parameters in tomato fruits (*p* < 0.05).

Treatments	Concentration of Sugars–Total Acidity	Concentration of Sugars–Concentration of Vitamin C	Total Acidity–Concentration of Vitamin C
Control (without treatment)	0.14	−0.47	0.48
Biological control (Nematophagin–Mycopro)	0	−0.97	0.48
Chemical control (Vydate 5G)	−0.97	−0.5	0.85
Fitoverm	−0.14	−0.98	0.94
Liquid culture *Metarhizium anisopliae* F-22BK/2	0.33	0.65	0.84
Liquid culture *Arthrobotrys conoides* F-22BK/4	−0.5	0.87	0.19
Liquid culture *Paecilomyces lilacinus* F-22BK/6	0.6	−0.8	−0.59
Liquid culture *Trichoderma viride* F-294	−0.43	−1.0	−0.38

## Data Availability

The original contributions presented in this study are included in the article. Further inquiries can be directed to the corresponding author.
